# A unique plasma microRNA profile defines type 2 diabetes progression

**DOI:** 10.1371/journal.pone.0188980

**Published:** 2017-12-04

**Authors:** Paola de Candia, Gaia Spinetti, Claudia Specchia, Elena Sangalli, Lucia La Sala, Annachiara Uccellatore, Silvia Lupini, Stefano Genovese, Giuseppe Matarese, Antonio Ceriello

**Affiliations:** 1 Department of Diabetology and Dysmetabolic Diseases, IRCCS MultiMedica, Milan, Italy; 2 Department of Molecular and Translational Medicine, University of Brescia, Brescia Italy; 3 Laboratory of Immunology, Institute of Endocrinology and Experimental Oncology, National Research Council (IEOS-CNR), Naples, Italy; 4 Department of Molecular Medicine and Medical Biotechnology, University of Naples “Federico II”, Naples, Italy; 5 Insititut d'Investigacions Biomèdiques August Pi i Sunyer (IDIBAPS), C/Rosselló, Barcelona, Spain; 6 Centro de Investigación Biomédica en Red de Diabetes y Enfermedades Metabólicas Asociadas (CIBERDEM), Barcelona, Spain; SERGAS and IDIS, SPAIN

## Abstract

A major unmet medical need to better manage Type 2 Diabetes (T2D) is the accurate disease prediction in subjects who show glucose dysmetabolism, but are not yet diagnosed as diabetic. We investigated the possibility to predict/monitor the progression to T2D in these subjects by retrospectively quantifying blood circulating microRNAs in plasma of subjects with i) normal glucose tolerance (NGT, n = 9); ii) impaired glucose tolerance (IGT, n = 9), divided into non-progressors (NP, n = 5) and progressors (P, n = 4) based on subsequent diabetes occurrence, and iii) newly diagnosed T2D (n = 9). We found that impaired glucose tolerance associated with a global increase of plasma circulating microRNAs. While miR-148 and miR-222 were specifically modulated in diabetic subjects and correlated with parameters of glucose tolerance, the most accentuated microRNA dysregulation was found in NP IGT subjects, with increased level of miR-122, miR-99 and decreased level of let-7d, miR-18a, miR-18b, miR-23a, miR-27a, miR-28 and miR-30d in comparison with either NGT or T2D. Interestingly, several of these microRNAs significantly correlated with parameters of cholesterol metabolism. In conclusion, we observed the major perturbation of plasma circulating microRNA in NP pre-diabetic subjects and identified a unique microRNA profile that may become helpful in predicting diabetic development.

## Introduction

Type 2 diabetes (T2D) is a complex metabolic disorder. Its landmark is hyperglycemia, secondary to an altered insulin production by ß cells of the pancreatic islets, a disability of body cells to properly respond to insulin or both.

In order to plan an adequate prevention of T2D, it is necessary to ascertain individuals at increased risk for the disease, possible through the development of diabetes risk score assessment questionnaires [1, 2], without laboratory testing. Through the evaluation of traditional biomarkers it is then possible to identify subjects who already show metabolic alterations, such as higher than normal blood glucose levels but not yet high enough to be diagnosed as diabetic [3]. In particular, subjects with normal or mildly increased fasting glucose but with significant glucose metabolic abnormalities are recognized by the use of a 2-hour post-load glucose of a 75 grams glucose tolerance test (OGTT) [4]. This preclinical phase, often referred to as “pre-diabetes” is a randomly long and variable prelude to frank disease, in which it is still unfeasible to make an accurate prediction of T2D development.

In search of novel biomarkers that may respond to this unmet medical need, one highly suitable source is represented by small non coding RNA, in particular microRNAs (miRNAs) that, released by most cells in the body, reach blood circulation in a very stable form, and may be used to assess cell activity at distance [5]. In the past years, blood circulating miRNAs have become the most promising biomarkers for the diagnosis, prognosis and therapeutic options of a variety of pathological conditions such as cancer [6–8], cardiovascular diseases [9, 10], liver pathologies [11, 12] and sepsis [13, 14], among others, reviewed in [5, 15]. Importantly, an increasing number of studies has been published reporting the quantification (mostly through Real Time qPCR) of miRNAs in blood (either plasma or serum) of subjects with diabetes: many of these studies aimed at identifying circulating miRNA modulation in chronic diabetic complications, reviewed in [16]. Instead, the reports on circulating miRNAs from subjects with impaired glucose tolerance (or pre-diabetic) are more sparse. Moreover, these studies are either limited to a pre-selected number of miRNAs [17, 18], or a single miRNA of interest [19]. The two studies that are not hypothesis-driven and consider all detectable circulating miRNAs in a discovery effort, evaluate circulating miRNAs in subjects with impaired glucose tolerance with no distinction between those who later progressed to frank diabetes and those who did not [20, 21].

Beside miRNA potential capability to predict diabetes development, the profile of circulating miRNAs may also provide with useful information about diabetes pathogenic mechanisms. Although the assessment of circulating miRNA cell origin is not yet feasible, it is conceivable that miRNAs dysregulated in pre-diabetic and diabetic individuals are produced by organs whose function is altered in T2D (mostly pancreas, liver and adipose tissue). It is important to point out here that miRNAs have been shown to play a role in glucose metabolism at the intracellular level. Indeed, the deficiency of Dicer (the enzyme responsible for miRNA maturation) in pancreatic β-cells leads to up-regulation of insulin transcriptional repressors, reduced insulin expression and secretion, loss of glucose homeostasis and diabetes development in mice [22, 23]. The effect of dysregulated post-transcriptional gene silencing (miRNA-mediated) on the onset of insulin resistance and T2D is still elusive but accumulating evidence are pointing at miRNAs as new therapeutic targets to ameliorate metabolic disorders, reviewed in [24].

Our study is the first to quantify circulating miRNAs in pre-diabetic individuals with the retrospective knowledge of subsequent diabetes outbreak, hence the possibility to identify differentially expressed miRNAs in diabetic “progressors” *versus* “non-progressors” individuals as prognostic parameters. Thus, our working hypothesis is that levels of specific miRNAs may vary across the diabetes spectrum and provide useful readout of metabolic imbalance/disease progression and insights into T2D pathogenic mechanisms.

## Materials and methods

### 2.1. Human subjects

All subjects are part of an observational study for the diabetes screening, based at IRCCS MultiMedica in Milan, Italy [Diabetes Prediction And Screening Observational Study (DIAPASON), protocol number 24/2012(153)]. They were included for having an intermediate/high risk of diabetes using the FINDRISC (Finnish Diabetes Risk Score), a questionnaire that takes into account age, gender, body mass index, drug consumption, family history of diabetes, diet and physical activity [25]. In particular, subjects selected for this study showed normal glucose tolerance (NGT, n = 9), impaired glucose tolerance (IGT, n = 9) and type 2 diabetes (T2D, n = 9). The group inclusion criteria was based on the results of a 75 grams oral glucose tolerance test, with NGT showing plasma glucose level <140 mg/dL; IGT individuals between 140 mg/dL and 199 mg/dL; and T2D >200 mg/dL, 2 hours after glucose administration, in accordance with the diagnostic criteria of the American Diabetes Association [26]. Even though there is a progressive increase in average BMI passing from NGT to IGT to overt T2D, the difference is not statistically significant (Table 1). Our study does not have to consider medication effects because all our T2D subjects are newly diagnosed and hence drug naïve. The IGT group can be further divided in two: progressors (P IGT, who did develop diabetes, n = 5) and non-progressors (NP IGT, who did not, n = 4), based on diagnosis of diabetes during the 12 months follow up.

**Table 1:** Clinical data.

	NGT		IGT		T2D		
	% males		% males		% males		p-value*
Gender	44		56		22		0.3
	mean	sd	mean	sd	mean	sd	p-value**
Age (years)	57.9	8.9	62.9	7.3	60.2	8	0.4
Weight (Kg)	67.2	12.7	71.7	13	78.3	17	0.3
Height (cm)	167.9	10.5	166.9	9.7	163.3	11.9	0.6
BMI (Kg/m2)	23.7	3.3	25.6	3.3	29.6	7.8	0.07
Waist (cm)	88.2	13	96.6	7.5	101	11.3	0.06
Fasting glucose (mg/dL)	81.8	9.1	99.6	8.4	106.8	17.9	**0.0009**
OGTT glucose (mg/dL)	94.7	26.2	162.8	21	231.2	32.6	**<0.0001**
HbA1c millimoles	36.8	2.2	44.6	3.4	46.4	6.3	**0.0002**
Basal Insulinemia	11.6	12.3	31.3	48.5	18.8	11.9	**0.03°**
HOMA index	2.6	3.2	7.7	11.8	5.1	3.6	**0.02°**
Triglycerides (mg/dL)	103.3	58	158.7	69.9	116.1	61.7	0.2
Total Cholesterol (mg/dL)	196.4	26	218.7	23.2	199	30.5	0.2
LDL Cholesterol (mg/dL)	116.2	18.5	132.7	28	121.9	30.3	0.4
HDL Cholesterol (mg/dL)	59.6	14.2	54.2	17.9	53.9	16.5	0.7
Microalbuminuria (mg/L)	8	7.1	76.1	150.4	7.8	5	0.07°
Endothelial Function (Endopath)	2.2	0.5	2.4	0.9	2.5	0.7	0.7

Means and standard deviations (sd) for the indicated clinical parameters in subjects with normal glucose tolerance (NGT), impaired glucose tolerance (IGT) and diabetes (T2D). Oral glucose tolerance test (OGTT) refers to glucose level measured 2 hours post load. The reported p-values refer to: *Fisher exact test; ** Anova; °Kruskal-Wallis test. In bold p-values<0.05.

### 2.2. Laboratory testing

Anthropometric measurements including height, weight and waist circumference were performed by standardized procedures. Plasma glucose level was quantified by Slein method using a Siemens analyzer (Munich, Germany); triacylglycerol and total cholesterol were measured by an automated enzymatic colorimetric test (Siemens); glycated hemoglobin (HbA1c) was detected by Tosoh’s analyzer (Tosoh, Tokyo, Japan), and insulinaemia level by a CentaurusXP analyzer (Siemens). Homeostasis model assessment for insulin resistance (HOMA-IR) was calculated as fasting plasma glucose (mg/dL) x insulin (mUl/L)/ 22.5. For miRNA study, venous blood was drawn from the above subjects in an ethylene di-amine tetra-acetic acid (EDTA) anticoagulant tube and centrifuged at 3000 rpm for 10 min. The upper supernatant (plasma) was then stored and frozen at -80°C till RNA extraction, that was performed at the same time for all samples starting from 200 μl.

### 2.3. miRNA quantification and analysis

Plasma RNA was extracted using miRCURY™ RNA Isolation Kits–Biofluids (Exiqon, Denmark). Serum/Plasma Focus microRNA PCR panels (Exiqon, Denmark) were used to quantify 179 miRNAs, known to be detectable in human blood. To control RNA extraction efficiency, 3 RNA spike-ins (UniSp2, UniSp4 and UniSp5) pre-mixed, each at different concentration in 100 fold increments were added. To confirm that the reverse transcription and amplification occurs with equal efficiency in all samples, one spike-in (UniSp6) was added in the reverse transcription reaction. Raw data are reported in S1 Table. Upon raw Ct value data collection, assays with several melting points (or with melting points deviating from assay specifications), assays with amplification efficiency below 1.6 or assays giving Ct values within 5 Ct values of the negative control samples, are all flagged and removed from the data set. After quality control assay selection, the average number of assays detected per sample was 162, and 90 miRNAs were detected in all samples. The stability of the average Cq value of the 90 co-expressed miRNAs was higher than any single miRNA in the data set as measured by NormFinder software [27] and hence the normalized relative quantities (NRQs) were calculated as follow: 2^^(average Ct-assay Ct)^ and log transformed. The Delta Ct between miR-23a-3p and miR-451a (used as a parameter of hemolysis) did not show any significant difference between NGT, IGT and T2D group.

### 2.4. Statistics

Categorical variables were compared among the three diagnostic groups using Fisher’s exact test. Continuous variables were compared by use of ANOVA or by means of Kruskal-Wallis test if they had a skewed distribution. Correlations were evaluated by Spearman correlation coefficient. For differential expression, miRNA NRQs were analyzed by unpaired t Test. All reported p-values were two-sided. A p-value<0.05 was considered statistically significant. Statistical analyses were performed with STATA 12 software (StataCorp. 2011. Stata Statistical Software: Release 12. College Station, TX: StataCorp LP).

### 2.5. Ethics statement

The use of plasma from the DIAPASON study for research purposes has been approved by the Ethics Committee of IRCCS MultiMedica in Milan, Italy. Written informed consent regarding study participation was obtained from all involved individuals.

## Results

### 3.1. Impaired glucose tolerance associates with global dysregulation of plasma circulating miRNAs

**Fig 1:**
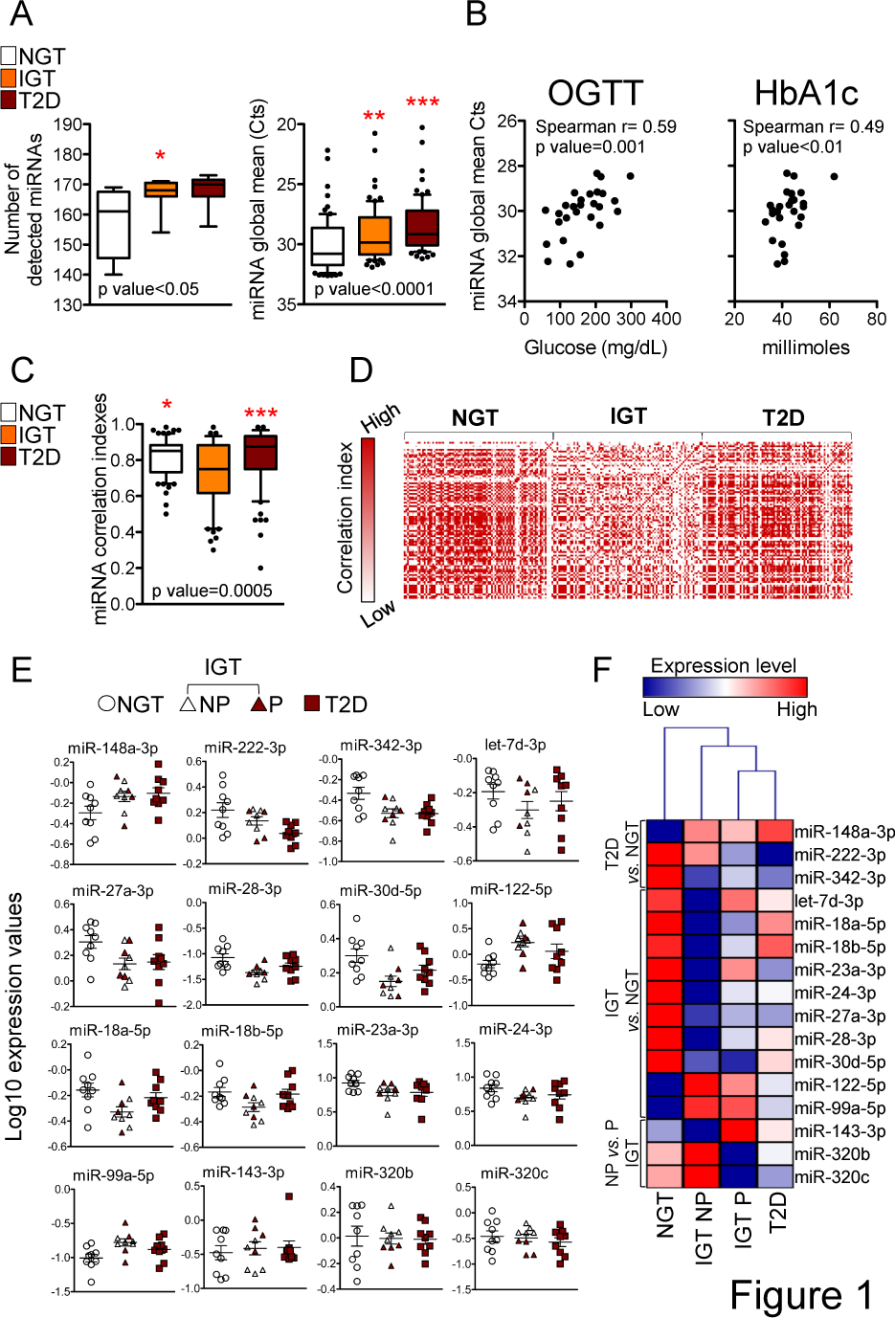
Dysregulation of plasma circulating miRNAs in glucose tolerance impaired subjects. (A) Boxplots (10–90 percentile) for the number of detected miRNAs (left) and miRNA Ct values (right) in the three indicated sample groups. Non-parametric Kruskal-Wallis p-values are reported at the bottom of the graph, while Dunn’s multiple comparison test (*versus* NGT group) p-values are reported on single group plots (* <0.05; ** <0.01; *** <0.001). (B) Scatter plots showing the correlation between miRNA global means (for the 27 individuals belonging to the three groups of NGT, IGT and T2D) expressed as reversed Ct values and either oral glucose tolerance test (OGTT, that refers to glucose level measured 2 hours post load, left) or glycated hemoglobin (HbA1c, right). Spearman correlation coefficient r and p-values are also reported. (C) Boxplot for single miRNA non-parametric correlation with the global mean values, averaged per group. Non-parametric Kruskal-Wallis p-value is reported at the bottom of the graph. Dunn’s multiple comparison test (*versus* IGT group) p-values are shown (* <0.05; *** <0.001). (D) Heatmap reporting the correlation index map (non parametric Spearman r values) of each co-expressed miRNA *versus* all the others. The map is divided per group as indicated. (E) Vertical scatter plots for Log10 transformed global mean normalized miRNA values in the three groups as indicated. The IGT group is divided in two further groups: progressors (P IGT) and non-progressors (NP IGT), based on their clinical history (see text). miRNAs were found differentially expressed in at least one group comparisons (t Test p-value<0.05, see Table 2). (F) Hierarchical clustering of the four groups (the IGT group being divided as in panel E) using the Log10 transformed normalized values of differentially expressed miRNAs (as for panel E).

In order to evaluate whether and how glucose tolerance impairment and development of diabetes affects the quantity of circulating miRNAs, we profiled 179 miRNAs (known to be easily detectable in human blood) in the plasma of 9 subjects with normal glucose tolerance (NGT), 9 with impaired glucose tolerance (IGT) and 9 with overt type 2 diabetes (T2D) (clinical characteristics are reported in Table 1, microRNA raw data are reported in S1 Table). On average, the number of detectable miRNAs increased when comparing NGT (miRNA n = 157.2±3.7) with IGT and T2D subjects (miRNA n = 166.9±1.7 and n = 168.1±1.7 respectively, Fig 1A, left panel). Ninety miRNAs were expressed in all samples. When focusing on these co-expressed miRNAs, their global quantity (i.e. miRNA Ct mean value) was shown to progressively increase in IGT and T2D compared to NGT subjects (Fig 1A, right panel). miRNA global quantity was also found to significantly correlate with glucose level measured upon a glucose tolerance test (OGTT) and with glycated hemoglobin (Fig 1B), suggesting that peripheral blood miRNA concentration may rise in conjunction with glucose tolerance impairment. We then evaluated whether glucose impairment impacts the level of all miRNAs uniformly or the observed miRNA global quantity increase changes the reciprocal relationships between miRNAs. Interestingly, individual miRNAs had a decreased level of correlation with miRNA global quantity and a decreased level of correlation with all the other miRNAs specifically in IGT compared to both NGT and T2D subjects (Fig 1C and 1D). This result suggested a “more chaotic intra-individual miRNA expression” or better a loss of quantitative co-regulation of circulating miRNAs in this group and led us to predict that the IGT group would contain the highest number of differentially expressed miRNAs. For differential expression analysis, we normalized single miRNA quantities by miRNA global mean (endogenous normalization factor) to eliminate inter-individual variance. miR-126-3p, which has been observed decreased in T2D plasma/serum in several studies [16, 19], was decreased in IGT and T2D compared to normal subjects in our cohort as well, although the difference did not reach statistical significance (data not shown).

### 3.2. A unique plasma circulating miRNA signature in non-progressor (NP) IGT subjects

We identified miRNAs differentially expressed in the group comparisons shown in Table 2 (t Test p-value<0.05). IGT group was considered as either a single group, or divided in two subgroups: the progressors (P IGT, individuals who had developed diabetes 12 months after sample collection) and non-progressors (NP IGT, individuals who had not). Overall, 13 miRNAs were found differentially expressed in either IGT or T2D subjects compared to NGT (Fig 1E and Table 2). The level of miR-148a-3p, miR-222-3p and miR-342-5p was found differential between NGT and T2D and intermediate in IGT. The majority of differentially expressed miRNAs, on the other hand, tended to be either elevated (miR-122-5p and miR-99a-5p) or decreased (miR-18a-5p, miR-18b-5p, miR-30d-5p, miR-23a-3p, miR-24-3p, miR-27a-3p, miR-28-3p) in IGT compared to NGT (Fig 1E and Table 2). Furthermore, the differential expression was still present when considering the NP IGT *versus* the NGT group for the majority of these miRNAs (8/9) but not when considering the P IGT group (2/9)(Table 2). None of the miRNAs differentially expressed in pre-diabetes and diabetes showed a significant modulation when subjects were stratified based on body mass index, showing that the differential expression that we observe associates with glucose metabolic imbalance and not with overweight per se (data not shown).

**Table 2:** miRNA differential expression.

	*versus* NGT				NP *vs*. P IGT
miRNA	All IGT	NP IGT	P IGT	T2D	
miR-148a-3p	n.s.	n.s.	n.s.	*	n.s.
miR-222-3p	n.s.	n.s.	n.s.	*	n.s.
miR-342-3p	*	*	n.s.	**	n.s.
let-7d-3p	n.s.	*	n.s.	n.s.	n.s.
miR-18a-5p	*	*	n.s.	n.s.	n.s.
miR-18b-5p	*	*	n.s.	n.s.	n.s.
miR-23a-3p	*	*	n.s.	n.s.	n.s.
miR-24-3p	*	n.s.	n.s.	n.s.	n.s.
miR-27a-3p	*	*	n.s.	n.s.	n.s.
miR-28-3p	**	**	n.s.	n.s.	n.s.
miR-30d-5p	**	*	*	n.s.	n.s.
miR-122-5p	**	***	*	n.s.	n.s.
miR-99a-5p	**	*	n.s.	n.s.	n.s.
miR-143-3p	n.s.	n.s.	n.s.	n.s.	*
miR-320b	n.s.	n.s.	n.s.	n.s.	*
miR-320c	n.s.	n.s.	n.s.	n.s.	**

t Test p-values of miRNAs differentially expressed in at least one comparison between subjects with normal glucose tolerance (NGT) and subjects with either impaired glucose tolerance (all IGT, non-progressors, NP IGT, and progressor, P IGT) or diabetes (T2D). The last three miRNAs are significant when comparing NP and P IGT subjects. p-values <0.05 *; <0.01 **, 0.001***.

When directly comparing progressors *versus* non-progressors, we identified miR-143-3p as being lower in NP IGT and miR-320b and miR-320c as being higher in NP IGT. The identified differentially expressed miRNAs were able to discriminate between NGT and either IGT or T2D subjects, as demonstrated by clustering analysis (Fig 1F). Moreover, P IGT were closely related to the T2D group, while the NP IGT group was more distant (Fig 1F), providing proof of a miRNA profile specifically defining the propensity to develop overt T2D.

### 3.3. Inverse correlation of IGT-modulated miRNAs with parameters of metabolic dysregulation

**Fig 2:**
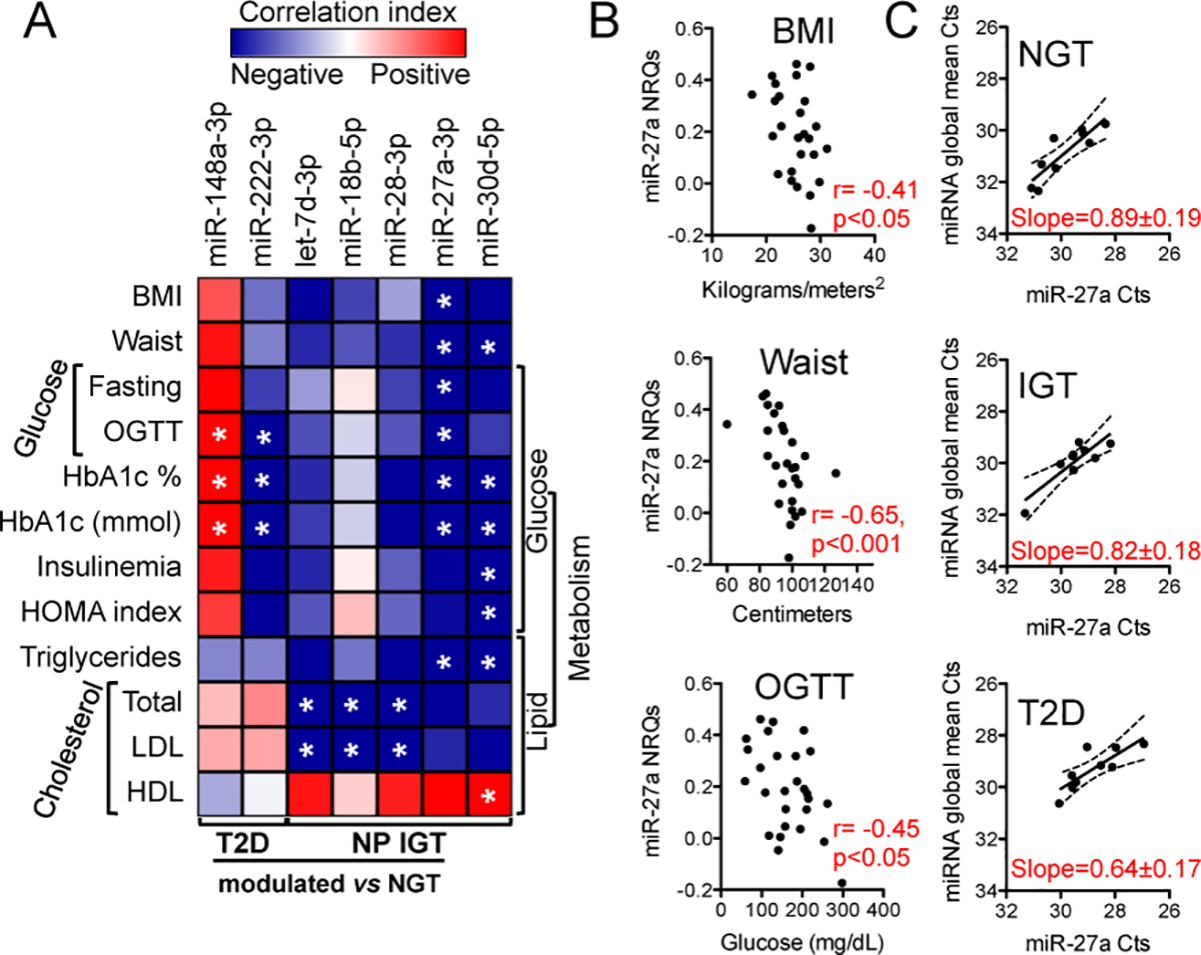
Analysis of miRNA correlation with metabolic parameters. (A) Heatmap reporting the correlation index map (Spearman r values, red if positive, blue if negative) of differentially expressed miRNAs and the reported clinical parameters. BMI = body mass index; OGTT, 2h oral glucose tolerance test, at 2 hours; HOMA = homeostatic model assessment; HbA1c = glycated hemoglobin; LDL = low density lipoproteins; HDL = high density lipoproteins. Only miRNAs significantly correlated with at least two clinical parameters are shown. * p-values≤0.05. (B) Scatter plots showing the correlation between BMI (upper), waist (middle) and OGTT (lower) and miR-27a-3p normalized relative quantities (NRQs) for all 27 subjects under study. Spearman r and p-values are reported. (C) Scatter plots showing the correlation between miR-27a-3p Ct values and miRNA global mean for all 27 subjects divided into the three groups of NGT, IGT and T2D as indicated. Slope values are also reported.

We then explored the relation between the identified differentially expressed miRNAs and clinical parameters of glucose and lipid metabolism. miR-148a-3p and miR-222-3p, found respectively increased and decreased in T2D subjects, were found to respectively positively and negatively correlate with OGTT and glycated hemoglobin (Fig 2A). miR-30d, which decreased in IGT *versus* NGT subjects was anti-correlated with both glycated hemoglobin and triglycerides (Fig 2A). Intriguingly, the group of let-7d-3p, miR-18b-5p and miR-28-3p all found to decrease in pre-diabetic subjects, negatively correlated with parameters of cholesterol metabolism (Fig 2A).

miR-27a, that was found to be low in IGT subjects, negatively correlated not only with parameters of glucose and lipid metabolism, but also with anthropometric measurements of BMI and waist (Fig 2A and 2B). Moreover, the correlation between the miR-27a raw Cts (“local parameters”) and miRNA mean Cts (global parameters) changed in the different groups of subjects, with the correlation slope becoming less steep in IGT and further less in T2D subjects compared to NGT (Fig 2C). This observation suggests that plasma circulating quantity of miR-27a, being significantly associated with clinical parameters of disease, becomes less and less dependent on the global miRNA quantity with disease progression.

## Discussion

By quantifying blood circulating miRNome in subjects with either normal or impaired glucose tolerance (NGT and IGT respectively) and diabetes (T2D), we found that disease progression associates with a remarkable increase of miRNA quantity per unit of plasma volume (i.e. global miRNA concentration). Our *ex vivo* approach does not allow to track down the cell origin of the observed miRNA increase, even though we hypothesize that it is produced by organs affected by T2D (pancreas, liver and adipose tissue for the most part). The increase of miRNA concentration may depend on an augmented number of miRNA releasing cells (such as adipose cells in over-weighted individuals), an enhanced miRNA release on a single cell basis (caused by an altered cellular activity in the course of disease), or a combination of both. Another fascinating possibility to be tested is that miRNA concentration may vary depending on an altered ratio between body weight and blood volume (i.e. volemia), potentially occurring in over-weighted individuals.

Before statistical analysis of differential expression, miRNA data normalization (based on the endogenous global miRNA level) is usually performed to reduce technical sample variation. It is important to underline that, being miRNA global quantity actually dependent on biological (not technical) variation in our sample set, this type of normalization process, by eliminating the difference in global quantity, led us to concentrate on how single miRNA expression varies in the different groups per unit of miRNA level. Nonetheless, we think that the potential biological effect of extracellular miRNAs on target cells may depend on both their actual concentration and relative quantity. Indeed, on one side the number of molecules of a microRNA is relevant for the impact that this microRNA can exert on a specific biological pathway or process when it is transferred from the extracellular space to target cells. On the other side, the reciprocal quantity relative to other microRNAs that are simultaneously transferred, and may display different or opposite effect on that specific biological pathway or process, is also important for the global effect of the extracellular microRNome in putative target cells.

Among the identified differentially expressed miRNAs, miR-27a represents the best example of miRNA modulation in condition of metabolic imbalance. This miRNA shows a unique behavior, seeming to be subject to two distinct forces: on one side, the above-mentioned general increase of all miRNAs with disease progression, on the other miR-27a tendency to be less expressed with higher measurements of BMI, waist, triglycerides, glucose and glycated hemoglobin. The result of these two forces is the detectable change of miR-27a correlation slope with miRNA global quantity, underlining the fundamental importance of data processing in this type of studies, especially in regard to the relative nature of qRT-PCR quantification.

In our study, more than in T2D, miRNA-miRNA relationships detectably changed in IGT subjects specifically, with a dramatic decrease of average correlation index between single miRNAs or between single miRNAs and global miRNA level. We have valued this phenomenon as potentially associated with a “more chaotic intra-individuals miRNA expression” in pre-diabetes. What may be the reason for that? IGT subjects are characterized by systemic glucose variability, marked by frequent hypoglycemic and hyperglycemic episodes. *In vitro* and animal studies, as well as observational investigation in humans, have demonstrated that multiple fluctuations of glycaemia may be more harmful and induce more deleterious effects in target tissues than a simple episode of acute hyperglycemia or, indeed, chronic stable hyperglycemia [28–30], suggesting that IGT condition may affect blood miRNA co-regulation more dramatically than frank diabetes. In line with this hypothesis, the majority of identified differentially expressed miRNAs were actually dysregulated in IGT more evidently than in T2D. The fact that miRNA expression was not substantially different in T2D subjects may be referred to the nature of our diabetic group, formed by individuals at onset for the disease, differently from other studies.

The major novel aspect of our study is the knowledge of who did and who did not progress to T2D among the IGT subjects, that led us to identify miRNAs able to classify non-progressors IGT *versus* progressors IGT and T2D. It is important to underline here that none of the clinical markers traditionally used as readout of glucose metabolism impairment (either fasting glucose and oral glucose tolerance test, or glycated hemoglobin) showed a differential level and/or was able to discriminate/clusterize the two IGT subgroups (not shown).

The level of blood glucose is subject to a plethora of environmental challenges, including diet regimen, and it has been proposed that miRNAs may help buffering variations in gene expression by counterbalancing stochastic perturbations and hence playing a role in glucose homeostasis [24]. Consistent with this hypothesis, some of the miRNAs that we have identified as differentially expressed in IGT compared to NGT subjects have been previously associated with glucose metabolism. miR-30d that we have found decreased in IGT condition, was described as down-regulated in the islets of obese diabetic leptin receptor deficient *db/db* mice. At a functional level, this miRNA is able to induce glucose-dependent insulin gene expression and to protect β-cells against TNF-α suppression of insulin transcription and insulin secretion, suggesting miR-30d may play multiple roles in preserving β-cell functions from the deleterious effects of pro-inflammatory cytokines [31, 32]. We have also identified miR-143 as significantly increased in IGT progressing *versus* non progressing to T2D subjects, and we reckon this result very intriguing. As a matter of fact, in mice, miR-143 transgenic overexpression was shown to decrease insulin sensitivity impairing glucose homeostasis, while miR-143 deficiency was shown to protect from obesity-associated insulin resistance [33]. In light of the evidence that dysregulated miRNA-dependent gene silencing actually contributes to the development of insulin resistance, our results open the way to the possibility that also at the extracellular level, blood-based miRNAs may contribute to glucose metabolic impairment. In other words, the pronounced dysregulation of blood circulating miRNAs observed by us in plasma of pre-diabetic subjects (in particular the under-representation of miR-30d and the differential expression of miR-143 in progressors *versus* non-progressors) may be part of a vicious cycle in which differentially expressed miRNAs are both an effect and a fuel of metabolic imbalance. Moreover, the coordinated modulation of miRNAs belonging to the same family (as in the case of miR-23-27-24 family member decrease in IGT) suggests the coherent behavior of a cluster of miRNAs may increase the impact on gene regulation and resultant biology in disease. Since blood level of liver-specific miR-122-5p has been repeatedly associated with dyslipidemia in both the human and murine system and also in pathological conditions other than diabetes [34–36], the significant uptick of miR-122-5p registered in our IGT subjects is most possibly to be ascribed to the average higher level of triglycerides, total cholesterol and LDL in this group compared to both NGT and T2D individuals (Table 1). Interestingly, increased levels of mi-122 has been recently linked to the improvement of the metabolic profile [37], suggesting that the validation of miR-122 further increase in non progressor pre-diabetic individuals may add important insight into the potential regulatory role of this miRNA in metabolic balance.

Finally, it is known that subgroups of microRNAs travel in blood also in association with high and low density lipoproteins [38]. Since we have performed RNA extraction from whole plasma, at this stage it is not possible to discern whether the correlation we observe between some miRNAs and either HDL or LDL depends on their direct association with or exclusion from these complexes in healthy and diseased conditions. In any case, these plasmatic miRNAs (let-7d-3p, miR-18b-5p, miR-28-3p and miR-30d-5p) deserve more attention for their potential ability to help cardiovascular prevention in subjects below the diagnostic threshold of diabetes.

## Conclusions

Our analysis has unexpectedly shown that the pre-diabetic glucose tolerance impairment, more than overt T2D onset, is associated with the major perturbation of plasma circulating miRNA level. When considering the published literature on circulating miRNAs in pre-diabetic and diabetic conditions, coherent results are unfortunately still scarce. The reason for that is to be attributed to many variables, including different source of samples, difference in population ethnicity, different pre-analytical and analytical procedures, the miRNA quantification platform and data normalization process [16]. Although this study sample size is very small, and the follow-up was only 12 months, it allowed us to identify a unique miRNA profile with the potential of predicting diabetic progression in the pre-diabetic population, that is worth of deeper investigation not only in larger sets of pre-diabetic but also in other metabolic syndrome diseased individuals. In the longer run, the standardization of operating procedures, across laboratory sample and data sharing and validation will become absolutely necessary to identify reliable and exclusive biomarkers.

## Supporting information

S1 Table microRNA quantification data. Ct raw data are reported as obtained from the Real Time qPCR experiment. Columns: plasma samples; rows: profiled microRNAs.
(TXT)
